# Effect of Internal Defects on the Fatigue Behavior of Additive Manufactured Metal Components: A Comparison between Ti6Al4V and Inconel 718

**DOI:** 10.3390/ma15196882

**Published:** 2022-10-03

**Authors:** Nicola Cersullo, Jon Mardaras, Philippe Emile, Katja Nickel, Vitus Holzinger, Christian Hühne

**Affiliations:** 1 Airbus Operations GmbH, 20355 Hamburg, Germany; 2 Institute of Mechanics and Adaptronics, Technische Universität Braunschweig, 38092 Braunschweig, Germany; 3 Airbus Operations SAS, 31060 Toulouse, France; 4 Institute of Composite Structures and Adaptive Systems, German Aerospace Center (DLR), 38108 Braunschweig, Germany

**Keywords:** Additive Manufacturing (AM), Laser Powder Bed Fusion (LPBF), Inconel 718, Ti6Al4V, internal defects, fatigue life prediction

## Abstract

In order to obtain a widespread application of Additive Manufactured (AM) technology in the aircraft industry for fatigue critical parts, a detailed characterization of the Fatigue and Damage Tolerance (F&DT) behavior of structural components is required. Metal AM techniques in particular are prone to internal defects inherently present due to the nature of the process, which have a detrimental effect on fatigue properties. In the present work, Ti6Al4V and Inconel 718 coupons with artificially induced defects of different dimensions were produced by the Laser Powder Bed Fusion (LPBF) technique. Fatigue tests were performed, and a different defect sensitiveness was observed between the two materials with Inconel being more defect tolerant compared to Titanium. The environmental role at the crack tip of internal defects was discussed, and based on a purely fracture mechanics approach, a simplified stress–life–defect size model was finally devised. The experimental test results together with the information obtained from the fracture surface analysis of tested samples are used to validate the model predictions. The proposed approach could be adopted to define a critical defect size map to be used for tailored Non-Destructive Testing (NDT) evaluation.

## 1. Introduction

In recent years, Additive Manufacturing (AM) processes—in particular Laser Powder Bed Fusion (LPBF)—have received a lot of attention in the aircraft industry. Compared to conventional manufacturing processes, the possibility of producing complex geometries with a higher freedom in the design, the efficient use of material and resultant mass reduction represent the main benefits that are leading to the adoption of AM components for aerospace applications. Despite the numerous advantages, to obtain a widespread application of the technology for fatigue critical parts, a detailed characterization of the Fatigue & Damage Tolerance (F&DT) behavior is required. Indeed, the entire production process needs to be reliably mastered in order to minimize process-induced characteristics such as defects or high surface roughness, which have a detrimental effect on fatigue properties [1]. Today, the adequate and robust F&DT quality of printed parts relies on the inhibitive constraint and discipline applied in the design and production phases. In addition, post-treatment processes, such as surface treatments and Hot Isostatic Pressing (HIP), and tight Non-Destructive Testing (NDT) requirements increase cost and lead time in manufacturing/production. NDT requirements in particular introduce additional limitations in terms of the part size that can be inspected, the geometry accessibility, and resolution to be adopted, which further affect the inspection cost. To relax the current requirements of post-processing steps and reliably transition toward defect-tolerant designs, an improved understanding of the effect of defects is required, which could lead to location-specific NDT. Internal stresses in every component are different, geometry features are not the same all over it, and therefore, the inspection size criteria need to be adopted on a local base approach. However, to simplify the part acceptance criteria process, a reliable and efficient modeling framework to calculate those tailored requirements is needed.

The present paper aims to make a step forward in the comprehension and relation of the effects of internal defects on the fatigue performance of AM parts, pointing out possible differences between two widely adopted materials in aerospace: Ti6Al4V and Inconel 718. On the one hand, the literature concerning effects of defects and fatigue performance of Ti6Al4V is rather extensive [2,3]; but on the other hand, Inconel 718 made by AM has been less widely studied. An attempt may be found in the work from Ogawahara et al. [4], where the authors investigated samples with internal defects and their influence on the fatigue strength, or in the one from Balachandramurthi et al. [5] where mainly the role of HIP was evaluated. Overall, although the effect of porosity on the mechanical behavior is widely discussed [6], a detailed and rigorous comparison of fatigue performance of parts produced by LPBF, both in Titanium and Inconel, subjected to internal defects, is not available in the open literature. Indeed, in most of the available research studies, defects are predominantly identified from fractographic observations, resulting in quite scattered data. For the above reasons, in the present work, an extensive and systematic test campaign is conducted to define a detailed comparison between different defect sizes at the fracture origin for different stress levels. In this way, a complete full characterization study on the influence of defects on the fatigue behavior of AM Inconel 718 and Ti6Al4V was obtained.

To devise a reliable prediction model and to investigate the relationship between the defect size, the local stress level and the fatigue life, different studies have been conducted. Various researchers worked to establish a relation between the fatigue limit and the defect size [7,8], including modeling of the fatigue behavior of AM components in the finite life regime following different approaches. Romano [9] proposed the use of the fatigue curve slope to factorize the El Haddad formulation for the fatigue limit region to any given finite life. Among different approaches, the use of a crack growth-based one was discussed by several authors who considered it as a promising and reliable method for the analysis of the fatigue life of AM components in respect to defects [10,11]. Ciavarella [12] proposed a new equation to generalize the Kitagawa–Takahashi diagram by interpolating the Basquin–Wöhler’s law for the uncracked material and the Paris integrated equation. Muhammad et al. [13] incorporate the projected defect size in the loading direction as initial crack together with the software NASGRO to determine the fatigue life of PBF stainless steel. A modified NASGRO model taking also into account the short crack growth was also adopted by Hu et al. [14], which provided an extended Kitagawa diagram involving the finite life region.

In the light of the above, the aim of the present work is not only to provide a detailed comparison toward the defects’ sensitivity on the fatigue behavior between two materials widely used in the aircraft industry but also to elaborate a practical engineering approach to easily determine the fatigue performance of AM components adopting a fatigue crack growth propagation model. It is worth noting that different types of defects may occur in 3D-printed parts (e.g., inclusions, gas pores, keyholes). Due to the procedure applied, the artificial defects obtained in the present work are comparable to lack of fusion.

## 2. Materials and Methods

### 2.1. Specimen Fabrication and Preparation

**Figure 1:**
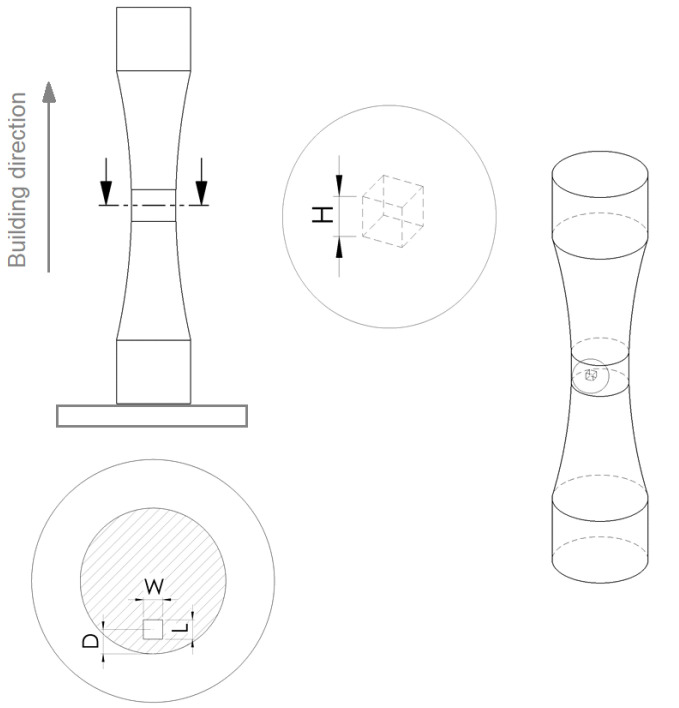
Schematic depiction of the coupon configurations on the build plate, including the induced artificial internal cubic defect.

The experimental activity of the present work is based on LPBF samples made by two different materials: Ti6Al4V and Inconel 718. Each set of samples, for the two different materials, was manufactured on a single machine with consistent process parameters. A commercially available printing system was utilized, and qualified standard process parameters were adopted. To avoid a possible anisotropic influence between the analyzed samples, coupons were printed in a single printing orientation. Figure 1 shows a schematic representation of the coupon nesting on the building platform.

**Figure 2:**
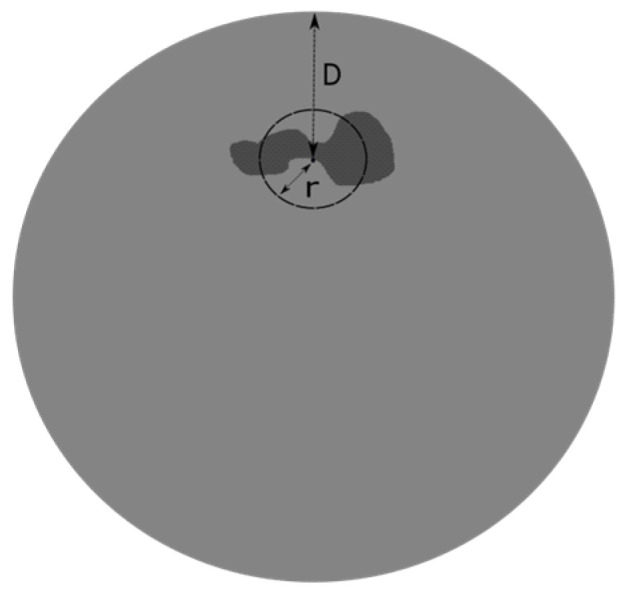
Equivalent defect of radius r at a distance D from the outer surface.

To investigate the influence of internal defects on the fatigue behavior of the two materials, artificial cubic defects were created in the specimens by selective non-melted areas (the internal defects were obtained by leaving a void in the CAD file, as shown in Figure 1). In particular, different defect configurations were investigated. The defect location relative to the closest outer surface was chosen according to an empirical rule introduced by Murakami [15]. According to the author, a defect may be considered internal if the following equation is verified:

(1) $$\frac{r}{D}<0.8$$

where *r* is the radius of the equivalent defect circle and *D* is the distance of the center of the defect from the closer external surface (see Figure 2).

For the sake of completeness and to define the ideal upper bound of fatigue strength to be expected in the absence of defects, samples with no artificial defects were also printed within the same print-batch. After the printing, all Ti6Al4V samples were subjected to a stress relieve at 720 °C, 2 h in a vacuum furnace. Then, samples followed an HIP process (920 °C and 1000 bar for 2 h), although without the pressure for the artificial defect coupons, aiming to not close the internal artificial defects but to achieve same final microstructure on all coupons. Heat treatment of Inconel samples with artificial internal defects was conducted in the following manner: (1) stress relieving at 1160 °C for 3 h, (2) solution treatment at 980 °C for 1 h in protected atmosphere/air quenched and (3) duplex annealing similar to AMS-5383 specifications. As completed for Titanium, Inconel samples with natural internal porosity were HIP treated; in step (1), an external pressure of 1020 bar was applied. Finally, to isolate the fatigue behavior of the samples with respect to internal defects, all samples were machined to minimize the possibility of a surface roughness failure initiation. A final roughness of $R_{a}<0.8\;\mu$m was achieved.

The different defects sizes tested are summarized in Table 1; defect dimensions are visualized in Figure 1. It has to be underlined that the defect size values reported in Table 1 represent the nominal ones: the ones planned for this study. It is worth mentioning that the defect dimensions values introduced in the CAD were a little bit bigger to obtain the desired nominal size. Regardless, all the failure surfaces were evaluated after the testing, and the real defect area was determined.

### 2.2. Fatigue Testing Condition

Axial fatigue tests were carried out on round coupons according to DIN EN 6072; to minimize variations in the stress state along the artificial defect, a geometry with a constant test diameter was chosen (similar to FCE type B, $K_{t}=1$). A resonance machine was adopted with a testing frequency between 110 and 155 Hz. All tests were performed at room temperature with a stress ratio R (min/max stress amplitudes) of 0.1. The maximum number of cycles was set at ten million. For each defect scenario given in Table 1, a full fatigue curve was determined performing at least two tests for each stress levels analyzed. The different stress levels were selected so to cover the fatigue life range between $10^{4}$ and $10^{7}$.

### 2.3. Fracture Surfaces Analysis

Upon completion of the fatigue tests, fracture surface investigations were carried out to confirm the validity of the crack initiation mechanism. A Keyence microscope was utilized. When the crack initiation was not clear, a Scanning Electron Microscope (SEM) was used to have a more accurate investigation of the fracture surface. In this way, the defect area that led to failure was determined.

## 3. Results

### 3.1. Fatigue Behavior and Defects at the Fracture Origin

**Figure 3:**
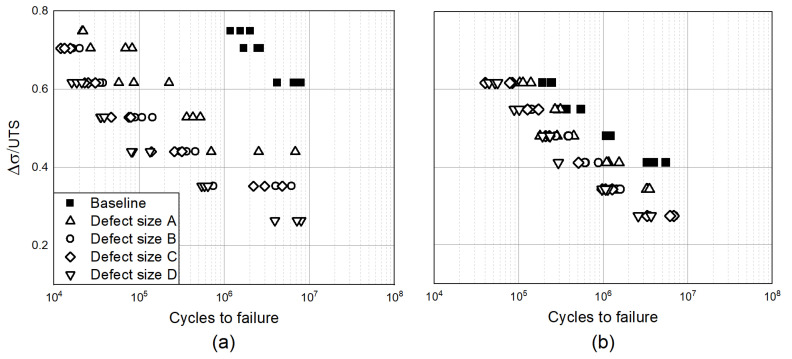
Experimental fatigue results of (**a**) Ti6Al4V and (**b**) Inconel 718: a comparison between different series of defect size at R = 0.1.

The fatigue test results for both Ti6Al4V and Inconel 718 samples are reported in Figure 3. All fatigue data have been normalized by the respective Ultimate Tensile Strength (UTS) value. The values adopted for the two materials were determined by quasi-static tensile tests according to DIN EN 2002-001:2005 performed on coupons printed and post-treated in the same conditions as the fatigue *Baseline* ones.

For both materials, the SN data points provided illustrate that the expected effect of the defect size was reproduced; larger defects result in decreased fatigue life. A clear and corresponding defect size influence on the lifetime is shown at all stress levels. This confirms the behavioral validity of the artificially induced defects obtained for each scenario.

**Figure 4:**
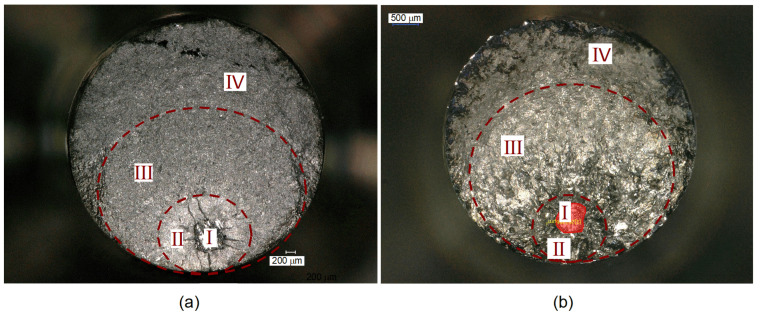
Characteristic fracture surface of Ti6Al4V (**a**) and Inconel 718 (**b**) coupons with a focus on the different crack propagation zones.

To characterize the crack initiation site, fracture surfaces were evaluated by the use of an optical microscope (see Figure 4). Figure 4a shows a characteristic fracture surface of a Ti6Al4V coupon with the artificial internal defect.

Four zones can be identified: (I) the crack initiation site where the artificial defect is clearly visible, (II) a bright circular area around the crack initiation site, (III) the main area where the stable crack propagation phase took place, and (IV) an area which indicates the final ductile fracture. The bright fish-eye circular zone is typically observed around internal crack initiation sites and serves as an indicator of the internal crack propagation [15]. As identified by Fomin [16], the fish-eye radius is equal to the distance from the internal defect to the surface. The smooth fracture surface causes a higher light reflection and the consequent bright appearance of the area. The explanation, as it will be discussed later in the paper, can be traced back to the absence of atmospheric effect within the first part of the defect propagation and its influence as soon as the defect becomes exposed to the external surface. The same observation can be deduced for Inconel coupons (see Figure 4b), where the environmental effect seems also to have an effect in the propagation area close to the defect, but it is less pronounced that in the Ti samples.

**Figure 5:**
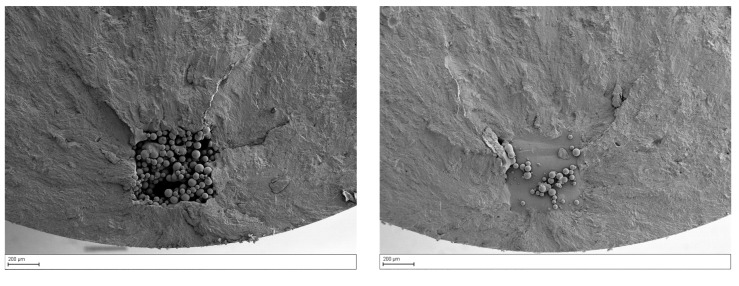
Typical failure location of Ti6Al4V samples at the first not melted layer. Top (**left**) and bottom (**right**) half on the same test coupon.

It should be noted that the crack initiation location (along the defect height) is quite different in the two materials. Indeed, in Titanium samples, the failure started at the first not melted layer of the artificial defect. An example is shown in Figure 5, where the two half bottom and top parts of the same sample are shown.

**Figure 6:**
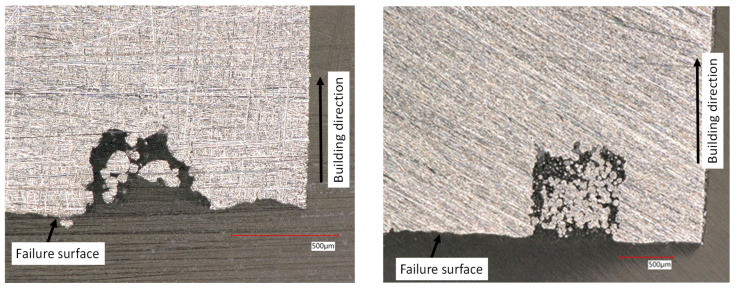
Cross-section of two different fatigue Ti6Al4V coupons in correspondence of the artificial defect. Failure occurred in both samples at the first not-melted layer.

The same mechanism was observed in all defect configurations. To understand the reason behind this different failure, two of the tested Ti6Al4V samples were cut in the middle in correspondence of the artificial defect (see Figure 6). The failure seems to occur at the first not melted layer due to the defect morphology. The maximum stress peak is expected in proximity of this area due to an immediate change in the cross-section or to the largest projected area. These represent the area with the highest stress concentration due to the sudden change in the stress field and so the transition into the critical area where the failure started.

**Figure 7:**
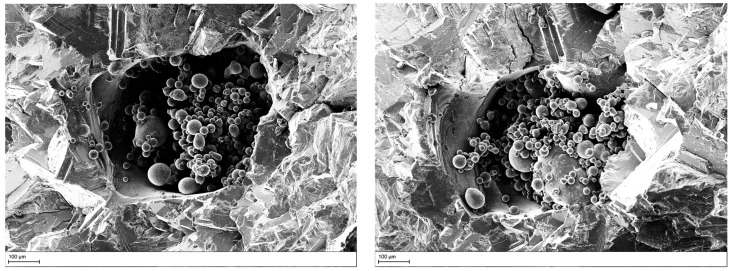
Typical failure location of Inconel 718 samples at the middle of the defect height. Top (**left**) and bottom (**right**) half on the same test coupon.

On the other hand, the failure in most Inconel 718 samples seems to start in the middle of the defect height (see Figure 7). Artificial defects in Inconel seem to be more round; edges are less sharp compared to the one observed in Ti6Al4V samples. For this reason, in contrast to what happened in the Titanium sample, the failure did not start from the first skipped melted layer of the defect but in the middle.

The observations outlined above indicate that it is indeed the largest section of the defect, perpendicular to the loading direction, which is driving the fatigue initiation location. Similar observations were made by Gong et al. [17]. In their work, fatigue specimens were printed with cylindrical and double conical internal defects with varying heights but a consistent area perpendicular to the building direction. No change in fatigue performance was observed across the samples, and fractography analyses confirmed consistent fracturing processes. To further explore that point, in the present work, the role of the defect height is also analyzed (see Section 3.3).

### 3.2. Material Comparison: Ti6Al4V and Inconel 718

**Figure 8:**
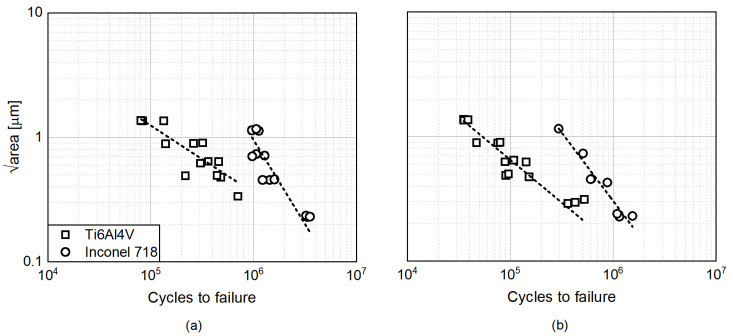
Influence of defects size identified by fractographic analysis on the cycles to failure for two distinct stress levels: (**a**,**b**).

In general, Inconel shows a higher tolerance for defects in terms of fatigue life, as can be seen in Figure 3. Ogawahara et al. [4] arrived at a similar conclusion in the study of Inconel samples with varying internal defects at a constant stress amplitude. In particular, they showed a negligible effect on the fatigue limit and the fatigue life of internal defects of different dimensions. On the other hand, the fatigue life of Ti6Al4V samples is more sensitive to internal defects. In the present work, to show the more detrimental impact of defects on the Titanium samples, the defect size influence on the cycles to failure for the different stress levels was evaluated. Two of them are here reported (see Figure 8). Here, the measured defect in terms of $\sqrt{area}$ parameter at the origin of the failure is employed (not the nominal one set in the initial CAD file and reported in Table 1).

As can be seen in Figure 8, the slope of the fitting curve is consistent for the two different stress levels (≈0.58 for Ti64 and ≈1.07 for Inconel 718). This shows reproducible results in terms of the defects’ impact on the fatigue performance for the different scenario. In particular, the slope identified in the Titanium results is roughly double that of the Inconel one. The higher slope of the latter shows a lower sensitivity of the material toward defect; the fatigue life decreases slowly if the defect size increases.

### 3.3. The Role of the Defect Height

To evaluate the influence of the defect height, two additional artificial defect configurations were tested (see Table 2). In this case, only Ti6Al4V samples were produced and tested. The same behavior was expected for Inconel.

**Figure 9:**
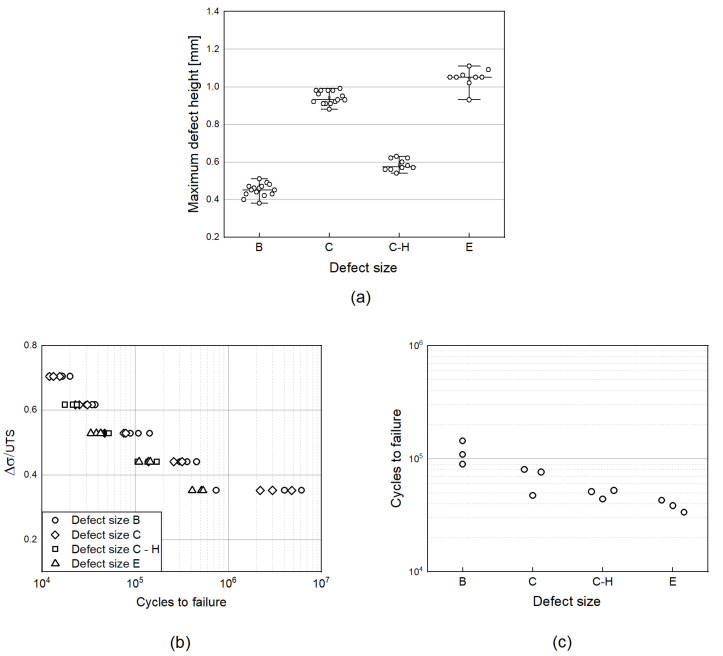
(**a**) Maximum defect height determined from CT scan analysis for the different defect size scenario coupons; (**b**) experimental fatigue results of Ti6Al4V samples with a focus on the effect of defect height; (**c**) cycles to failure for the different defect scenario for a given stress level.

The defect height for every scenario was computed by μ-Computed Tomography inspection. Data analysis and evaluation was performed with the help of the software VG Studio Max (Volume Graphics GmbH). The maximum defect height recorded for each sample is reported as a function of the defect size in Figure 9a. A comparison with the defect scenario analyzed in the previous sections is highlighted. The corresponding fatigue results with a focus on the effect of defect height are reported in Figure 9b,c. In particular, the latter shows the cycles to failure of the different configurations for a given stress level (the same results may be obtained if the other stress levels are used). As identified in Figure 9a, the artificial defects in Defect size C and C-H have similar dimensions perpendicular to the loading direction but different defect heights. The defect height in scenario C-H is close to that in scenario B. On the other side, for scenario E, a bigger defect area perpendicular to the loading direction is expected compared to scenario C, but a similar defect height was measured.

The fatigue results show that the key parameter is the largest dimension perpendicular to the loading direction, while the defect height plays only a secondary role. Indeed, the fatigue behavior of scenarios C and C-H are similar and worse than that of scenario B. The results are also confirmed by scenario E, where an increase of the dimension perpendicular to the loading direction led to a decrease of the fatigue performance. To conclude, from the experimental results obtained, the assumption made that cracks initiate at the widest cross-sectional area of the defect is suitable. In reality, results also show that although the influence is not so relevant, a small trend seems to suggest that the lower the height, the lower the fatigue performance. Future work will be carried out to evaluate this aspect, but overall, it can be concluded that since the variation in the fatigue performance is so small that it can be neglected, the major role is played by the dimension perpendicular to the loading direction.

## 4. Stress-Life-Defect Size Modeling

A simplified fatigue prediction model based on a crack growth approach is here presented. In particular, the aim of the authors is to obtain an analytical solution to be adopted for NDT requirements to achieve a target fatigue performance. In the present section, the fatigue life of the tested coupons is estimated using a fracture mechanics-based approach taking as input the internal defects analyzed by fractographic analysis. To determine the defect size, the area of the measured defect was adopted. This represents the area of the projected defect size on the failure plane perpendicular to the loading direction.

### 4.1. Kitagawa Diagram for Infinite Life

**Figure 10:**
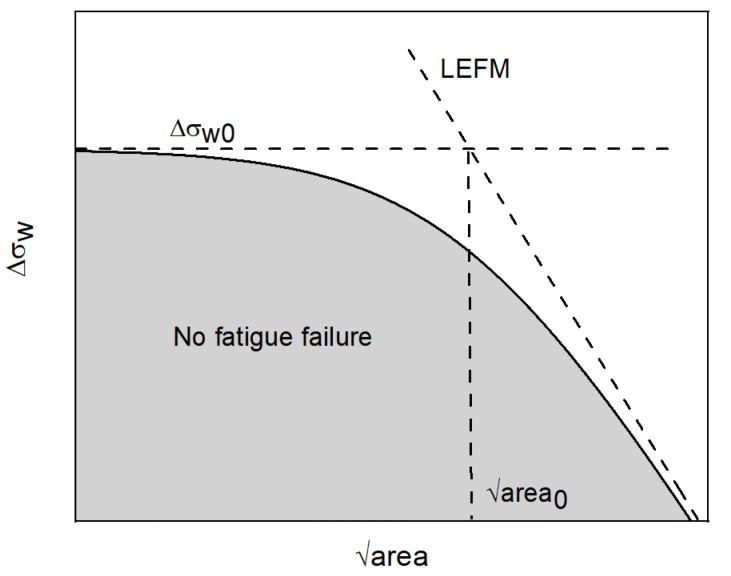
Schematic representation of the Kitagawa diagram.

Defects may be analyzed in terms of stress raisers or according to Murakami treated as short cracks. To show the similarity between defects and small cracks for the fatigue limit assessment, different models were developed (Murakami [18], Chapetti [19], El-Haddad [20]). These models may be used to describe the different parts of the Kitagawa–Takahashi diagram [21], one of the most effective tools to obtain, for given cracks length or defect area, the allowable stress range for infinite life (see Figure 10).

Generally, when dealing with fracture mechanics, the energy that makes the crack propagate is the key parameter that needs to be evaluated. This energy may be expressed by the Stress Intensity Factor (SIF). According to Murakami, who proposed a well-established method to estimate the size of irregular defects [18], the SIF may be determined according to the following equation

(2) $$\Delta K=Y\Delta \sigma \sqrt{\pi \sqrt{area}}\;$$

where Δσ is the stress range applied to the defect, $\sqrt{area}$ is the projected area perpendicular to the loading direction, and *Y* represents the influence of the defect position as well as defect shape depending on the formulation adopted. According to Murakami, the value of Y may be set to 0.5 for embedded defects and 0.65 for surface one (i.e., a surface defects leads to a 30% higher SIF than an internal defect).

As mentioned, different models can be used to describe the Kitagawa diagram. Among them, the El Haddad model is accurate and relatively simple, requiring only two material parameters: the fatigue limit $\Delta \sigma_{w0}$ and the fatigue crack growth threshold $\Delta K_{th,LC}$. Keeping the SIF expression used before (2), the relation between fatigue limit and defect size may be expressed as

(3) $$\Delta \sigma_{w}=\Delta \sigma_{w0}\sqrt{\frac{\sqrt{area_{0}}}{\sqrt{area}+\sqrt{area_{0}}}}\;$$

where $\Delta \sigma_{w0}$ is the fatigue limit in absence of defects and represents an upper limit for a given microstructure. The expression $\sqrt{area_{0}}$ is called the El-Haddad parameter

(4) $$\sqrt{area_{0}}=\frac{1}{\pi }{\left(\frac{\Delta K_{th,LC}}{Y\Delta \sigma_{wo}}\right)}^{2}\;$$

where $\Delta K_{th,LC}$ is the fatigue crack growth threshold for long cracks. The advantage of introducing this parameter is to have a smooth transition between the short crack and the long crack regime. The same graph shown in Figure 10 may be represented in terms of $\Delta k_{th}$ following the expression:

(5) $$\Delta K_{th}=\Delta K_{th,LC}\sqrt{\frac{\sqrt{area}}{\sqrt{area}+\sqrt{area_{0}}}}$$

Following such an approach, if the stress level is below the identified curve, defects should not propagate.

**Figure 11:**
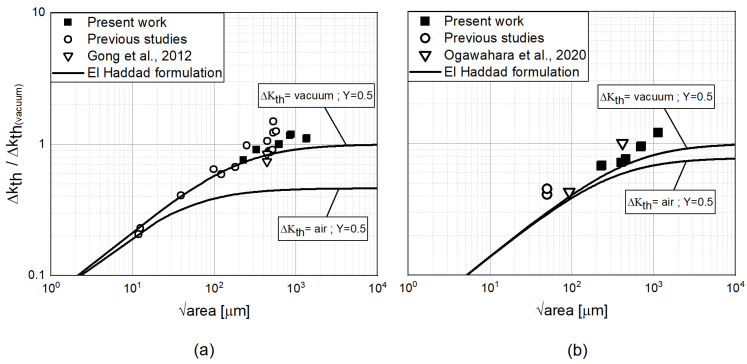
Kitagawa diagram for both Ti6Al4V (**a**) and Inconel 718 (**b**) showing experimental data points that survived more than one million cycles. Data points from literature were also used (Gong et al., 2012 [17], Ogawahara et al., 2020 [4]).

To define the El-Haddad model, the parameter $\Delta K_{th,LC}$ for long cracks is needed. For the present work, no tests were performed; hence, no data about the $\Delta K_{th,LC}$ were available for the two materials adopted. To overcome this limitations and to populate the Kitagawa diagram in the infinite life regime, test data from several works available in literature were considered together with the experimental tests data here analyzed and data from previous studies. In particular, for the present analysis, experimental points that survived more than one million cycles were reported in the diagram (see Figure 11).

**Figure 12:**
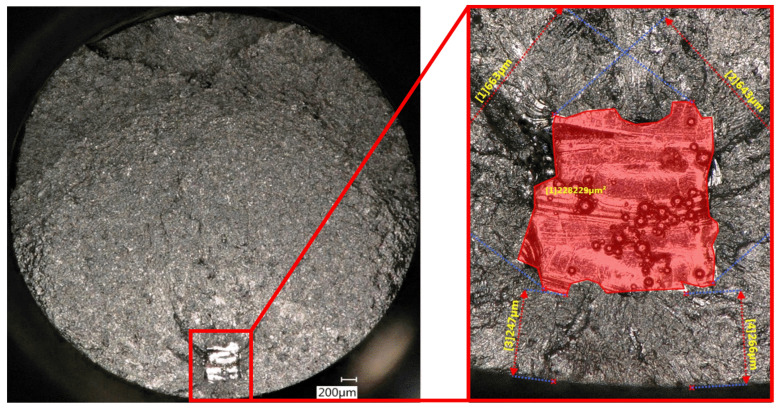
Defect area determination through fractographic analysis.

The defect area was determined by identifying a smooth defect contour in the fractographic analysis performed (see Figure 12).

It is worth noting that the $\Delta K_{th}$ identified for the Titanium material to match the data points is nearly a factor of two compared to the one determined by Wycisk [3] or adopted by Fomin [16] ($\Delta K_{th}=air$). The main reason for this higher threshold value might be attributed to the environmental effect on the internal defects analyzed. The environmental effect on the fatigue damage mechanism was analyzed by several authors. In particular, for Ti6Al4V, Junet [22] confirmed the results previously obtained by Yoshinaka [23]: the crack growth rate of internal cracks is lower than cracks initiated from the surface. The real environmental conditions at the crack tip of internal cracks is not known, and it represents still an open point, but it is clear that it has an influence on the crack growth rates [24]. In particular, Nakamura et al. [25] used synchroton radiation micro CT images to measure the internal crack growth rate and compared it with that of a surface crack in vacuum. Plotting the da/dN versus ΔK points, they demonstrated that the propagation rate of the internal crack was similar to the one in vacuum, but once the internal crack has reached the surface, it changes its propagation behavior to that of a crack directly exposed to the surface. From the above outcome, it is clear that the main influence between the crack propagation of internal and surface crack is coming from the surrounding environment. Buffiere et al. [26] in agreement with Nishijima [27] showed that the internal crack seems to propagate in a vacuum-like environment. So, internal crack growth rates should be deduced from tests performed in vacuum. In reality, as also suggested by Junet [22], another possibility would be to directly study the effect of internal defects. The latter approach was the one followed in the present analysis. Although the Ti6Al4V coupons adopted by Yoshinaka [23] were not obtained by AM, it is worth noting that the $\Delta K_{th}$ value for a long crack in vacuum is approximately the one adopted in the El-Haddad equation shown in Figure 11 ($\Delta K_{th}=vacuum$) and that has been used to normalized all data points. Similar values were also obtained by Irving [28] and McClung [29].

Contrary to Ti6Al4V, only a few studies regarding the propagation threshold in vacuum were conducted for Inconel. Nevertheless, if from one side, in Ti6Al4V samples, a remarkable slow propagation rate and a higher $\Delta K_{th}$ is evident [27,30], on the other side, as suggested by [31,32], the threshold values in air compared to the one in vacuum seem closer for Inconel 718. This result is partially confirmed by the fact that the $\Delta K_{th}$ value identified by the El-Haddad approach (see Figure 11b $\Delta K_{th}=vacuum$) is consistent with the results obtained by FCG test in air from Yadollahi [10].

**Figure 13:**
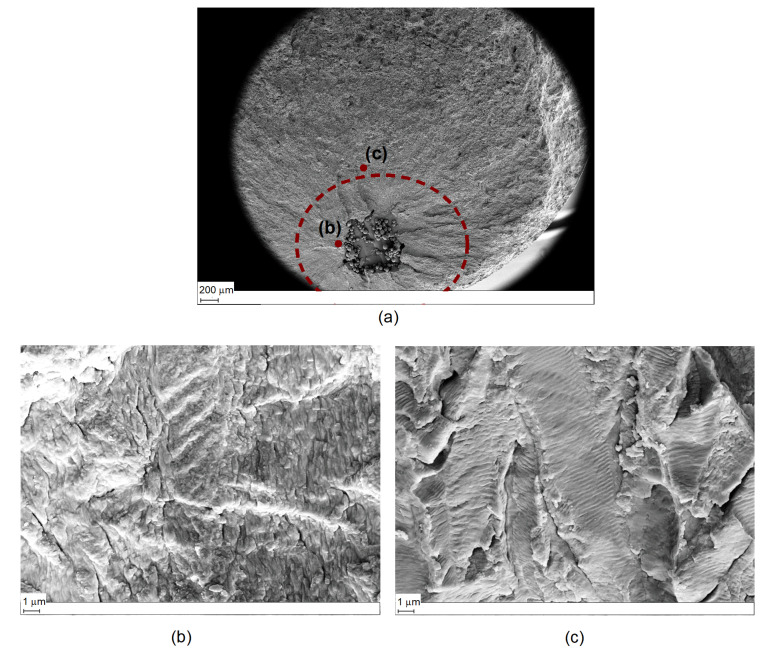
(**a**) SEM picture from failure surface of a Ti6Al4V coupon; (**b**) fracture surface near the artificial defect in the region around ΔK=12 MPa$\sqrt{m}$; (**c**) fracture surface far form the artificial defect in the region around ΔK=25 MPa$\sqrt{m}$.

To verify the different defect propagation in Ti6Al4V, SEM analysis was conducted on one of the artificial defect configurations (see Figure 13a). Figure 13b shows a region close to the defect in the propagation Stage II (the bright circular area identified in Figure 4). The fracture surface may be led back to the one observed in vacuum propagation with more round edges and with granular features, as also highlighted by Yoshinaka [23]. On the other hand, a smoother appearance can be observed in Figure 13c, where a region in Stage III is shown. The fracture propagation that is supposed to happen in air during this stage shows a clear striation patterns which was not identified in Stage II. This confirms the fact that a different propagation took place. The same result was obtained in [23] where no striations were formed in vacuum.

If the environment at the crack tip of internal cracks corresponds to vacuum or not is not fully clear; nevertheless, the fact that internal cracks propagate at lower rates helps to understand why usually failure starts from surface defects. The results obtained clearly suggest that this effect should be taken into account for the fatigue life prediction. In the present work, although the defect is supposed to start in a vacuum-like environment, the threshold regime needs to be corrected; after a certain number of cycles and before the failure, the crack becomes exposed to the surface and propagates in air. For this reason, for the long crack regime, data from FCG tests conducted in air will be adopted.

### 4.2. Extended Kitagawa toward Finite Life: A Fatigue Crack Growth Approach

In practical airframe applications, designs toward finite life are usually performed. To extend the Kitagawa curve toward the finite life regime, an approach based on crack growth is here proposed.

#### 4.2.1. Crack Propagation Modeling

**Figure 14:**
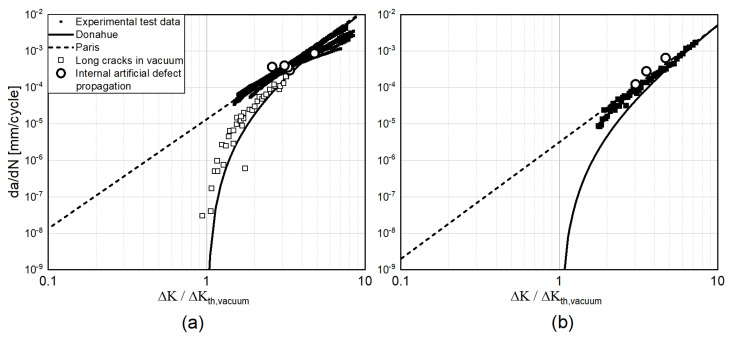
Fatigue crack growth data for (**a**) Ti6Al4V and (**b**) Inconel 718. The $\Delta K_{th}$ values identified for the two materials through the Kitagawa diagram are, respectively, highlighted in the two graphs. The Titanium propagation data in vacuum were taken from [33].

Fatigue crack growth experiments to obtain the reference crack propagation data (da/dN vs. ΔK) were performed using standard Compact Tension (CT) specimens. Tests were performed at R = 0.1. Results are shown in Figure 14. The experimental points were taken using several CT coupons with a width (W) = 40 mm and a thickness (B) = 10 mm obtained from materials subjected to the same heat treatment as for the coupons evaluated. As a reference for Ti6Al4V, the fatigue crack propagation data in a vacuum-like environment reported by Oguma [33] are also shown. All test data have been normalized by the $\Delta K_{th}$ value in vacuum adopted in Figure 11.

As already discussed, no striations were identified in the Stage II propagation of the analyzed coupon. On the contrary, clear striation patterns were observed once the defects became exposed to the surface. Several points were reported for both materials that follow the experimental test data obtained from crack propagation in air.

To model the crack propagation behavior, different approaches may be adopted. The most commonly used due to its simplicity is the Paris law:

(6) $$\frac{da}{dN}=C{\left(\Delta K\right)}^{m}$$

This is generally used to describe the intermediate regime of the crack propagation curve, since it neglects both the threshold region and the fracture toughness part. To take into account the threshold regime, an early attempt was made by Donahue et al. [34]:

(7) $$\frac{da}{dN}=C{\left(\Delta K-\Delta K_{th}\right)}^{m}$$

The Donahue equation is a simplified model compared to the commonly adopted NASGRO [35] relationship that covers both the initial low growth rate and the final increased rate. Nevertheless, neglecting the unstable crack growth part does not result in a significant error, since it represents a negligible portion of the total life [36]. Moreover, the aim of this work is to define a simplified approach and determine an analytical relationship between defect size, stress and fatigue life. For this reason, the Donahue solution was adopted in the present paper.

#### 4.2.2. Model Hypothesis

To model the stress–life–defects size relationship, the Donahue equation described in the previous section is integrated from an initial crack (or defect) size to its final dimension before failure:

(8) $$N=\int_{a_{i}}^{a_{f}}\frac{da}{f(\Delta K,\Delta K_{th})}$$

**Figure 15:**
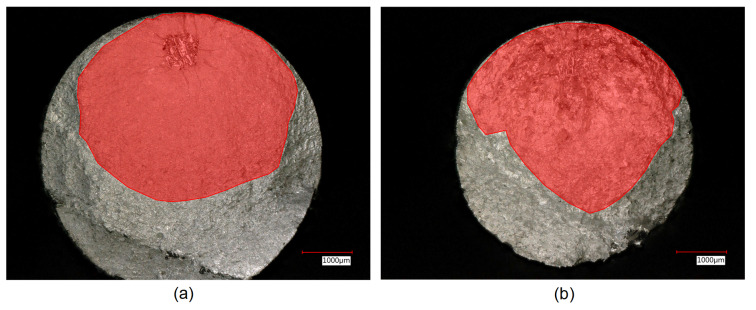
Final failure area before ductile fracture for (**a**) Ti6Al4V and (**b**) Inconel 718.

To solve the integration and to obtain an analytical solution, appropriate simplifications need to be taken into consideration:

- The geometrical factor Y is chosen following the Murakami binary approach (0.5 for the present analysis, since all the defects are internal). Although the Y factor increases approaching the surface, the factor is then kept constant in the integration. It has to be underlined that the geometrical factor depends not only on the defect position but also on the defect shape. For the sake of simplicity, defects were considered as a circular shape. The effect of aspect ratio (a/c) with a and c, the two biggest defect dimensions, is considered to remain constant during the propagation and therefore was not accounted for. From the fracture surface observations, the crack seems to propagate uniformly in all directions from the artificial defects; therefore, considering a shape ratio equal to 1 can be accepted. It is worth noting that this hypothesis was confirmed by Junet et al. [22], who performed in situ synchroton X-ray tomography during fatigue tests, observing that internal cracks after initiation propagate with a regular and almost circular shape.
- The integration of Equation (8) requires the initial and the final crack lengths. The initial $a_{i}$ defect size is determined using the $\sqrt{area}$ parameter obtained from the projected size perpendicular to the loading direction. In the present work, this was determined by fractographic observations, as shown in Figure 12.
- The final crack size can be either determined from the toughness value or from fractographyc analysis. In the present work, it was estimated by the fracture surface observations performed (see Figure 15). The final area before the ductile fracture was determined for all the samples, and an average value was used to determine the equivalent final crack dimension. It is worth mentioning that this value does not affect noticeably the final result because most of the propagation life happens at smaller crack sizes.
- Due to the misconception of using long crack $\Delta K_{th}$ for small defects [21,37], short crack $\Delta K_{th}$ was evaluated by means of the El-Haddad formulation in Equation (9); the value is then kept constant in the integration.
  (9) $$\Delta K_{th}=\Delta K_{th,LC}\sqrt{\frac{a_{i}}{a_{i}+a_{0}}}$$

#### 4.2.3. Model Validation

**Figure 16:**
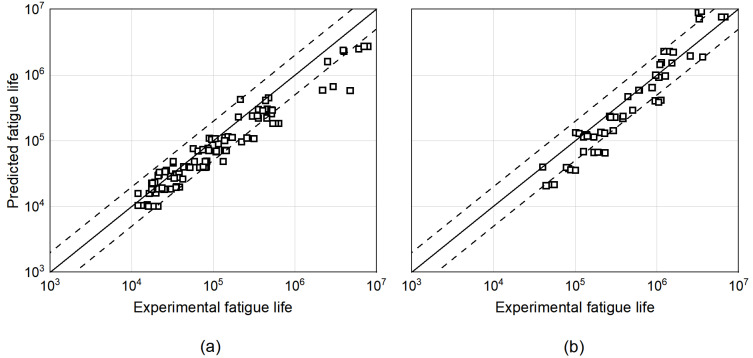
Results of FCG-based model fatigue life correlation to the experimental fatigue life obtained for Ti6Al4V (**a**) and Inconel 718 (**b**).

The experimental results presented in Section 3 are used to validate the model. The defects’ dimensions were characterized by the fractographic analysis performed, and their size was used as the initial flaw for the prediction model. Figure 16 shows the fatigue life correlation between the estimated fatigue lives and experimental results for all the different defect scenarios for both materials. The black line provides the perfect fit between the two results, while the dotted lines represent the scatter bands of a factor of two. According to several authors [38,39], a method works sufficiently well if the predicted points lies within this range.

From the results obtained, it has to be underlined that for both materials, most of the points fall into the scatter band, and in particular, for most of them, a conservative prediction is obtained. Moreover, it can be observed that for almost all samples outside the scatter band (e.g., Titanium samples in the high fatigue life regime), a conservative prediction is given. Overall, it can be concluded that despite the simplified approach, the proposed FCG model correlates well with the experimental test data in the area of interest.

## 5. Conclusions

The present study summarizes the research activity aimed at evaluating the influence of internal defects on the mechanical performance of AM Ti6Al4V and Inconel 718 samples produced by LPBF. Artificial internal defects of different dimensions were produced to evaluate the impact on the fatigue behavior. The results indicate a different behavior in terms of defect sensitivity of the two materials toward internal defects. This suggests the need to define different defect acceptance criteria in serial production depending on the material adopted. Moreover, in the present work, a relationship between stress–life–defect size has been proposed. Despite the simplifications adopted, it can be concluded that the fracture mechanics-based model described shows promising results, and it can support the application of AM aerospace components in structural areas. However, it has to be underlined that the proposed methodology was validated using only internal defects; hence, only the effect of internal anomalies was taken into account. The same model needs to be validated for defects closer to the surface or directly exposed to it. Moreover, also the influence of the surface roughness was avoided by the performed surface machining. For the latter, the same approach might be used if an Equivalent Initial Flaw Size (EIFS) based on roughness parameters can be established [40].

To sum up, the proposed approach may be used to define a critical defect size map to be used for tailored NDT evaluation. That is, given an in-service stress (typically based on structural analysis) and a target design life, an allowable defect size determined for different zones may be obtained. It is worth mentioning that such a model might be potentially adopted for quick evaluations during in situ monitoring to identify potential critical locations leading to a quality control procedure during the printing process.

## Figures and Tables

**Table 1:** Defect scenario configuration; dimensions are reported in μm. The baseline scenario represents the HIP-treated samples with the normal porosity level obtained from the process adopted.

ID	Defect Configuration (W × L × H)	Number of Samples	
		Ti6Al4V	Inconel 718
Baseline	Natural defects	9	12
Defect size A	200 × 200 × 200	14	14
Defect size B	500 × 500 × 500	15	15
Defect size C	750 × 750 × 750	15	15
Defect size D	1200 × 1200 × 1200	15	15

**Table 2:** Defect scenario configuration for Ti6Al4V samples with a variation in the defect height. Dimensions are reported in μm.

ID	Defect Configuration [W × L × H]
Defect size C-H	750 × 750 × 500
Defect size E	1000 × 1000 × 750

## Data Availability

Not applicable.
